# Optical emission from focused ion beam milled halide perovskite device cross‐sections

**DOI:** 10.1002/jemt.24069

**Published:** 2022-02-03

**Authors:** Felix U. Kosasih, Giorgio Divitini, Jordi Ferrer Orri, Elizabeth M. Tennyson, Gunnar Kusch, Rachel A. Oliver, Samuel D. Stranks, Caterina Ducati

**Affiliations:** ^1^ Department of Materials Science and Metallurgy University of Cambridge Cambridge UK; ^2^ Center for Convergent Technologies Istituto Italiano di Tecnologia Via Morego, 30 Genoa 16163 Italy; ^3^ Cavendish Laboratory University of Cambridge Cambridge UK; ^4^ Department of Chemical Engineering and Biotechnology University of Cambridge Cambridge UK

**Keywords:** cathodoluminescence, energy conversion and storage, energy harvesting, focused ion beam milling, perovskite, solar

## Abstract

Cross‐sectional transmission electron microscopy has been widely used to investigate organic–inorganic hybrid halide perovskite‐based optoelectronic devices. Electron‐transparent specimens (lamellae) used in such studies are often prepared using focused ion beam (FIB) milling. However, the gallium ions used in FIB milling may severely degrade the structure and composition of halide perovskites in the lamellae, potentially invalidating studies performed on them. In this work, the close relationship between perovskite structure and luminescence is exploited to examine the structural quality of perovskite solar cell lamellae prepared by FIB milling. Through hyperspectral cathodoluminescence (CL) mapping, the perovskite layer was found to remain optically active with a slightly blue‐shifted luminescence. This finding indicates that the perovskite structure is largely preserved upon the lamella fabrication process although some surface amorphisation occurred. Further changes in CL due to electron beam irradiation were also recorded, confirming that electron dose management is essential in electron microscopy studies of carefully prepared halide perovskite‐based device lamellae.

**Research Highlights:**

- Cathodoluminescence is used to study the emission of focused ion beam milled perovskite solar cell lamellae.
- The perovskite remained optically active with a slightly blue‐shifted luminescence, indicating that the perovskite structure is mostly preserved.

## Graphical abstract

Normalized perovskite emission spectra from (red) top‐view PL, (orange) top‐view cathodoluminescence (CL), (green) first cross‐sectional CL scan, and (blue) second cross‐sectional CL scan. As the signal‐to‐noise ratio for the second cross‐sectional CL scan is relatively low, normalization was performed on the Gaussian fit (blue line) rather than on the data points.

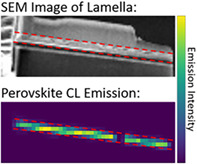

The past decade has witnessed the rise of optoelectronic devices based on organic–inorganic hybrid halide perovskites, including solar cells, photoelectrochemical cells for solar fuel production, and energy‐efficient light‐emitting diodes (Kim et al., 2020; Liu et al., 2021; Singh et al., 2020). Our understanding of their fundamental properties, working principles, and degradation mechanisms at the nanoscale has been advanced through transmission electron microscopy (TEM) characterization (Hidalgo et al., 2019; Ran et al., 2020). TEM is one of only a few techniques capable of probing the nanoscale heterogeneities in perovskite devices, making it invaluable as perovskite compositions and device architectures get more complex over time (Kim et al., 2020). Due to the multi‐layered nature of those devices, many TEM studies used focused ion beam (FIB) milling with gallium ions (Ga^+^) to prepare the electron‐transparent cross‐sectional lamellae (Divitini et al., 2016; Jeangros et al., 2016). However, the suitability of FIB milling for specimen preparation of beam‐sensitive halide perovskite has been questioned due to the expected Ga^+^ beam‐induced heating and irreversible surface amorphisation through accumulation of defects created by ion collision cascades (Baba et al., 1997; Rothmann et al., 2017; Rothmann et al., 2018). Therefore, FIB‐induced modifications to halide perovskite lamellae need to be understood to guide future characterization work performed on them and determine the validity of those in the literature.

The structural integrity of perovskite in FIB milled lamellae can be probed by observing its radiative emission as the intensity and energy of perovskite luminescence are dependent on its crystal structure. Briefly, several groups have shown that compressing various halide perovskites induces gradual amorphisation. This causes a continuous reduction in photoluminescence and a band gap widening of ~0.2 eV, until emission is eventually eliminated by strong non‐radiative recombination in the fully amorphous state (Wang et al., 2015; Wang et al., 2016; Wang et al., 2017; Zhang et al., 2017; Zhu et al., 2018). The band gap widening was found to be caused by suppression of atomic orbital overlap due to pressure‐induced breaking of long‐range order in the perovskite lattice (Wang et al., 2015; Zhu et al., 2018). Interestingly, the original crystallinity and luminescence were largely recovered when the pressure was relaxed (Wang et al., 2015; Wang et al., 2016; Wang et al., 2017; Zhang et al., 2017; Zhu et al., 2018). The observed relationship between perovskite structure and luminescence means the latter can be used as a proxy to examine the former. This link between the two properties is valuable to assess the suitability of FIB milling since amorphisation is the primary form of damage suspected in FIB milled perovskite lamellae due to ion collisions. A perovskite lamella producing an emission peak that is centered at the same energy as its parent device's emission can be taken as a sign that the perovskite's crystalline structure in the lamella is not amorphized by FIB milling. A lamella with a partially amorphized perovskite layer is expected to exhibit a broadened and blue‐shifted luminescence peak relative to its parent device. Finally, an absence of emission is considered a manifestation of complete perovskite amorphisation.

In this work, we show through hyperspectral cathodoluminescence (CL) measurements that the perovskite layer in FIB milled perovskite solar cell (PSC) lamellae remains luminescent with an emission that is blue‐shifted by 0.08 eV compared to a reference top‐view CL spectrum. We attribute this blue‐shift to partial surface amorphisation by the Ga^+^ beam and further amorphisation by the CL electron beam. However, the fact that the perovskite is still emitting strongly indicates that its crystal structure is largely intact. Finally, we found that PbI_2_ emission was isolated to a few small areas in the perovskite layer, suggesting minimal perovskite decomposition to PbI_2_.

We prepared a triple cation, double halide perovskite half‐cell with a stack of glass/tin‐doped indium oxide (ITO)/NiO/Cs_0.05_FA_0.81_MA_0.14_Pb(I_0.9_Br_0.1_)_3_. We first acquired top‐view PL and CL data from this half‐cell as reference points to evaluate the perovskite emission. A peak emission at 1.596 ± 0.002 eV (777 nm) and 1.612 ± 0.004 eV (769 nm) was observed for PL and CL, respectively (Figure 1, red and orange curves). The small blue‐shift from PL to CL and the high‐energy tail seen in the CL spectrum are often observed in literature, with both usually attributed to the filling of higher energy states by the higher concentration of excited carriers in CL (Cortecchia et al., 2017; Dar et al., 2016; Riesen et al., 2019). We fabricated a full‐cell device with a phenyl‐C_61_‐butyric acid methyl ester (PCBM)/bathocuproine (BCP) electron transport layer and gold electrode, then cut a 200 nm‐thick lamella using a Ga^+^ FIB. This lamella thickness has previously been identified as the ideal value for TEM characterization of FIB milled perovskite device cross‐sections by Jeangros et al. (Jeangros et al., 2016) This lamella was attached to a TEM grid and immediately transferred to the CL instrument, limiting air exposure to <5 min to prevent environmental degradation (Jeangros et al., 2016). The CL data was then post‐processed and fitted with a Gaussian model in LumiSpy (Ferrer Orri et al., 2021). Further details on the FIB milling procedure, CL data acquisition, and data processing are available in the Supporting Information S1.

**FIGURE 1:**
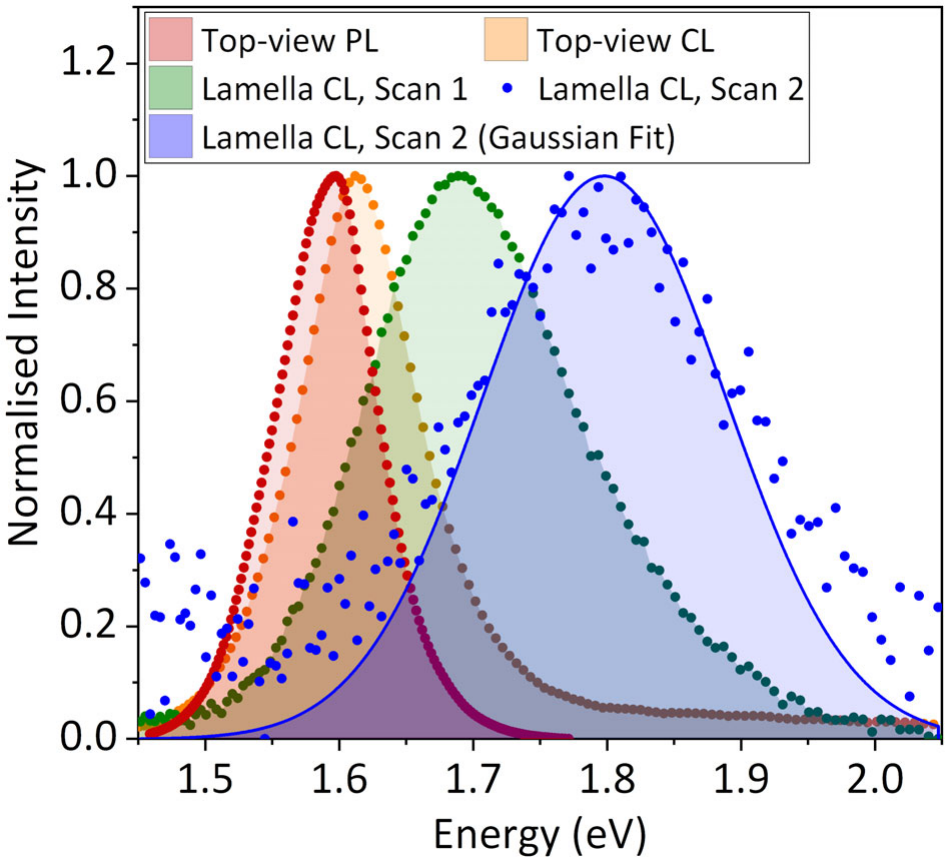
Normalized perovskite emission spectra from (red) top‐view PL, (orange) top‐view CL, (green) first cross‐sectional CL scan, and (blue) second cross‐sectional CL scan. As the signal‐to‐noise ratio for the second cross‐sectional CL scan is relatively low, normalization was performed on the Gaussian fit (blue line) rather than on the data points

Figure 2a shows a secondary electron image of the lamella, acquired with a beam acceleration voltage of 5 kV. Figure 2b,c show the fitted peak center energy and integrated peak area of the perovskite emission, respectively, for the first CL scan. The interaction volume generated by the 5 kV electrons spans the entire 200 nm thickness of the lamella, as indicated by the Monte Carlo‐based electron trajectory simulations shown in Figure S1 (Drouin et al., 2007). This suggests that the CL emission emerges from the entire lamella thickness, with the top half contributing approximately two‐thirds of the total intensity (Figure S1).

**FIGURE 2:**
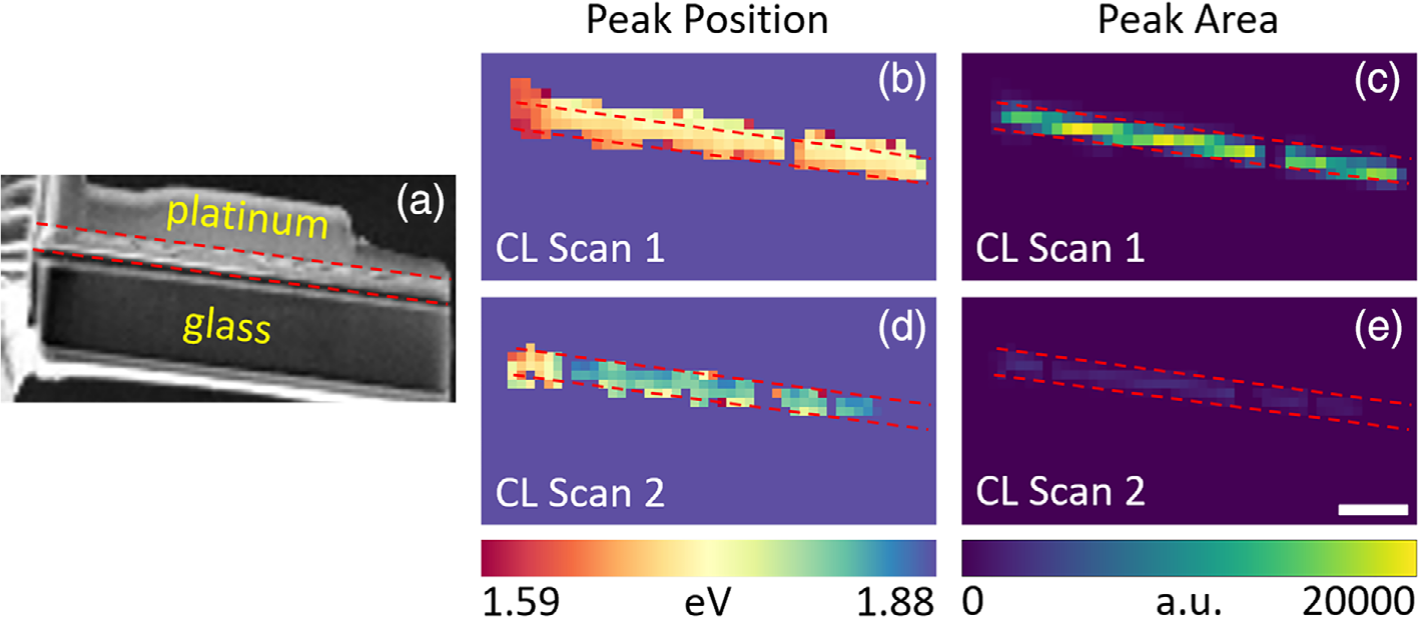
Perovskite emission characteristics from the (b,c) first and (d,e) second cross‐sectional CL scans of a FIB milled PSC lamella. (a) Secondary electron image, (b,d) fitted peak emission energy, and (c,e) peak emission area. Dashed red lines mark the position of the perovskite layer. Scale bar represents 2 μm and applies to all panels

Figure 1 (green curve) and Figure 2b,c show that the perovskite layer in the FIB milled lamella is luminescent, suggesting that the crystal structure is mostly preserved. Its emission is centered at 1.688 ± 0.004 eV (734 nm) with a full width at half maximum (FWHM) of 0.185 ± 0.006 eV, blue‐shifted by 0.076 ± 0.006 eV (35 nm) and broadened by 0.078 ± 0.008 eV compared to the top‐view CL spectrum (FWHM = 0.107 ± 0.006 eV, orange curve in Figure 1). The blue‐shifted and broadened perovskite emission indicates that only a fraction of the perovskite, most likely the closest to the lamella's top and bottom surfaces, is amorphized by the Ga^+^ beam. While the emission data do not directly provide structural information, amorphisation is the most common type of specimen damage in Ga^+^ FIB milling, especially for semiconductors (Giannuzzi et al., 2005; Huh et al., 2013; Liu et al., 2020; Mayer et al., 2007; Pastewka et al., 2009; Salvati et al., 2018), and it is also consistent with the observation of blue‐shifted emission from amorphized perovskite as described above (Wang et al., 2015; Wang et al., 2016; Wang et al., 2017; Zhang et al., 2017; Zhu et al., 2018). This amorphisation is due to defect accumulation, thus it and the consequent emission blue‐shift are irreversible, in contrast to the reversible pressure‐induced amorphisation (Wang et al., 2015; Wang et al., 2016; Wang et al., 2017; Zhang et al., 2017; Zhu et al., 2018). In addition, a small extent of iodine volatilization, which could be anticipated as it is more volatile than bromine, may also contribute to the blue‐shift (Kosasih et al., 2020).

While it is established that a Ga^+^ beam induces amorphisation, a low‐energy electron beam (4.5–60 eV) can also cause specimen damage in halide perovskites (Milosavljević et al., 2016). Therefore, it is of interest to know whether the observed changes in emission are solely caused by the Ga^+^ beam or by the CL electron beam as well. The effect of the CL electron beam on lamellae cannot be properly assessed by comparing the PL and top‐view CL spectra because of the different nature of bulk specimens and thin lamellae. For example, the higher specific surface area of a lamella likely accelerates the loss of volatile molecules, a known beam damage mechanism in hybrid halide perovskites (Chen et al., 2018; Chen et al., 2020; Rothmann et al., 2018). To obtain an estimate of the effect of the CL electron beam on the lamella, we performed another CL scan on the same lamella and extracted its fitted emission parameters (Figure 2d,e) and average spectrum (Figure 1, blue dots). The perovskite layer was barely optically active after this second scan, with its emission weakened by a factor of eight, broadened (FWHM = 0.213 ± 0.006 eV), and further blue‐shifted by 0.110 ± 0.006 eV (44 nm) compared to the first scan (Figure S2). These results suggest that the CL electron beam contributes to perovskite amorphisation, in good agreement with previous studies which observed CL emission darkening and blue‐shifting by 0.10–0.25 eV after exposure to an electron beam (Hentz et al., 2016; Xiao et al., 2015; Yuan et al., 2016). Therefore, the 0.076 ± 0.006 eV blue‐shift observed between the top‐view CL and first cross‐sectional CL spectra was likely caused by both the Ga^+^ beam and the CL electron beam (see also Supplementary Note S1).

In addition, we observe PbI_2_ emission (2.41–2.46 eV) only from a small number of isolated areas in the perovskite layer in both cross‐sectional CL scans (Figure 3). PbI_2_ has previously been identified as a product of beam damage‐induced perovskite decomposition (Xiao et al., 2015). However, the presence of a PbI_2_ peak in the top‐view CL spectrum (Figure S3) suggests that the 4% excess lead salts in the perovskite precursor solution is the most likely source of the PbI_2_ emission observed in Figure 3, as opposed to Ga^+^ beam‐induced perovskite decomposition.

**FIGURE 3:**
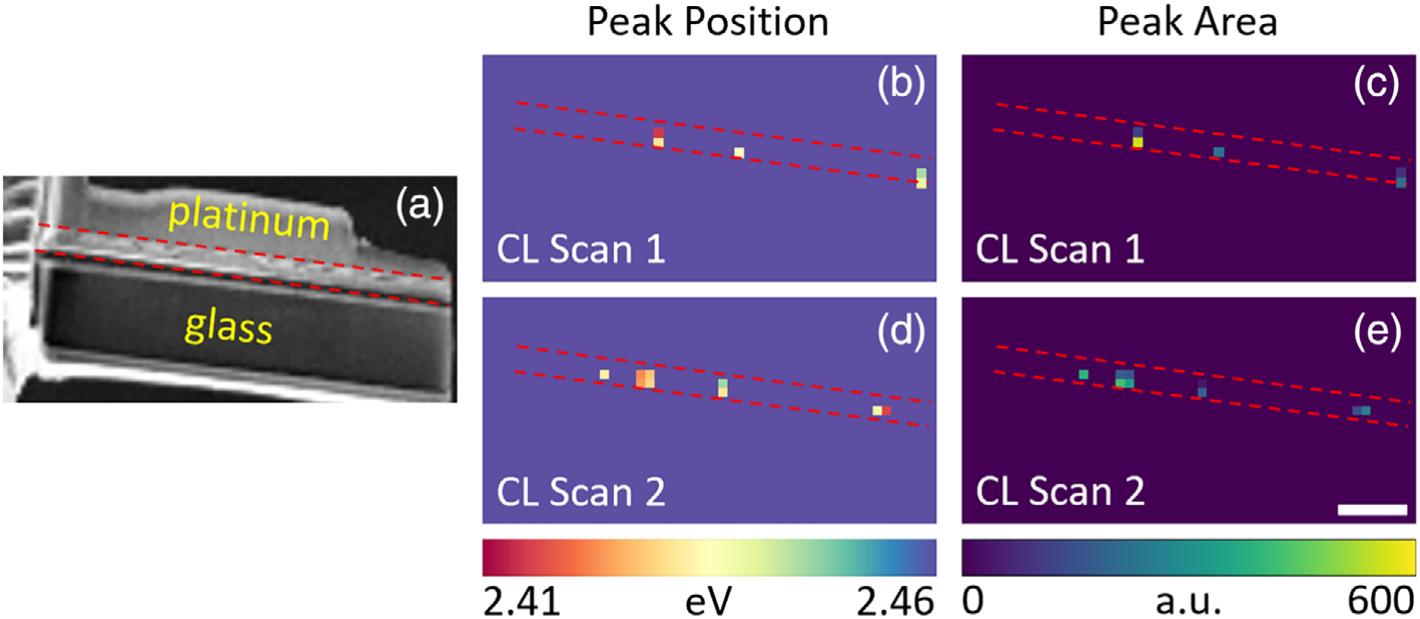
PbI_2_ emission characteristics from the (b,c) first and (d,e) second cross‐sectional CL scans of a FIB milled PSC lamella. (a) Secondary electron image, (b,d) fitted peak emission energy, and (c,e) peak emission area. Dashed red lines mark the position of the perovskite layer. Scale bar represents 2 μm and applies to all panels

In conclusion, we show that a typical FIB milled PSC lamella remains optically active, albeit with a slightly blue‐shifted luminescence compared to its top‐view CL emission. This blue‐shift supports the idea that PSC lamellae do not perfectly represent their parent device in terms of radiative emission. However, the extant luminescence and limited PbI_2_ emission indicate that the perovskite structure and composition are in large part preserved. Hence, useful information can be obtained from electron microscopy studies of FIB milled perovskite‐based device lamellae as long as electron dose is minimized and beam‐induced damage is carefully considered. For example, investigations of device morphology, compositional heterogeneity, or comparative studies of multiple lamellae prepared with identical FIB milling parameters are fruitful methods of device characterization by transmission electron microscopy. Lastly, we note that lamella thinning optimization and technological advances in FIB milling, such as cryo‐FIB and the use of different ions, are likely to further limit specimen damage (Rivas et al., 2020).

## CONFLICT OF INTEREST

Samuel D. Stranks is a co‐founder of Swift Solar, Inc.

## Supporting information


**Appendix S1**: Supporting Information.

## Data Availability

The data that support the findings of this study are available from the corresponding author upon reasonable request.
