# Supplementary material for: Optical emission from focused ion beam milled halide perovskite device cross‐sections
Source: Microsc Res Tech. 2022 Feb 3;85(6):2351–5. doi: 10.1002/jemt.24069 (PMC9304233; doi:10.1002/jemt.24069)
Supplement: Supplementary file 1 — Appendix S1: Supporting Information. [file JEMT-85-2351-s001.docx]

Supporting Information

Optical Emission from Focused Ion Beam Milled Halide Perovskite Device Cross-sections

Felix U. Kosasih^#^, Giorgio Divitini^#^, Jordi Ferrer Orri^#^, Elizabeth M. Tennyson, Gunnar Kusch, Rachel A. Oliver, Samuel D. Stranks, Caterina Ducati*

Dr. F.U. Kosasih, Dr. G. Divitini, J. Ferrer Orri, Dr. G. Kusch, Prof. R.A. Oliver, Prof. C. Ducati

Department of Materials Science and Metallurgy

University of Cambridge

27 Charles Babbage Road

CB3 0FS

United Kingdom
E-mail: [cd251@cam.ac.uk](mailto:cd251@cam.ac.uk) (Prof. C. Ducati)

Dr. G. Divitini

Istituto Italiano di Tecnologia

Via Morego 30, Genoa, Italy

J. Ferrer Orri, Dr. E.M. Tennyson, Dr. S.D. Stranks
Cavendish Laboratory

University of Cambridge

JJ Thomson Avenue

CB3 0HE

United Kingdom

Dr. S.D. Stranks

Department of Chemical Engineering and Biotechnology

University of Cambridge

Philippa Fawcett Drive

CB3 0AS

United Kingdom

^#^These authors contributed equally

**Experimental Methods**

**Device fabrication**

The nickel oxide ink was prepared by adding 35.5 mg of NiCl∙6H_2_O to 1 mL of 2-methoxyethanol. After adding 20 µL of nitric acid the solution was heated at 75°C for two hours. The ink was aged for at least 2 days before use. The perovskite triple cation ink (Cs_0.05_FA_0.81_MA_0.14_Pb(I_0.9_Br_0.1_)_3_) was prepared by adding 1521.8 mg of PbI_2_, 104.3 mg of PbBr_2_, 44.8 mg of CsI, 479.1 mg of FAI and 55.0 mg of MABr to 1.899 mL of DMF and 0.601 mL of DMSO. The formulation contains a 4% excess of lead salts, and a DMF:DMSO volume ratio of 3.16:1. 50 µL of BMITFB were added to 1 mL of DMF, and 20 µL of this solution was added to 1 mL of the perovskite ink. Electron transport layer solution was prepared by adding 27 mg of PCBM to 750 µL of chlorobenzene and 250 µL of dichlorobenzene, and by adding 5 mg of BCP to 10 mL of isopropanol. All precursors were used as purchased.

Glass/ITO substrates were scrubbed with water and soap solution (Hellmanex 2% in deionised water) and cleaned with three stages of ultrasonic bath: first in water and soap, then in ultrapure water, and finally in isopropanol. After drying they were treated for 15 min in a UV/O_3_ tool (Novasonic). The nickel oxide ink was spun at 4000 RPM for 30 s and annealed for 5 min at 75°C, 10 min at 120°C and one hour at 300°C. After cooling down, the samples were transferred into a nitrogen-filled glovebox. The perovskite ink was spun at 4000 RPM for 35 s, and 180 µL of chlorobenzene were dropped after 20 s. The film was annealed for 10 min at 100°C. For the full device sample, PCBM was spun at 1700 RPM for 30 s and annealed at 100°C for 5 min. BCP was spun at 4000 RPM. Finally, a 100 nm-thick gold layer was deposited by thermal evaporation.

**Cross-sectional lamella preparation**

The cross-sectional PSC lamella was FIB milled using an FEI Helios Nanolab Dualbeam FIB/SEM. Milling was done using a Ga^+^ beam whose parameters are detailed in Table S1. This lamella was immediately transferred into an Attolight CL-SEM, minimising air exposure to ~5 min. After thinning, the lamella was not imaged using an electron or ion beam to prevent any additional specimen damage.

**Table S1.** FIB milling procedure and beam parameters for cross-sectional lamella preparation

| Step | Description | Beam | Voltage (kV) | Current (pA) |
| --- | --- | --- | --- | --- |
| 1 | Platinum deposition with electron beam | Electron | 5 | 1400 |
| 2 | Platinum deposition with ion beam | Ga^+^ ion | 30 | 280 |
| 3 | Trench milling | Ga^+^ ion | 30 | 2800 |
| 4 | Lamella surface cleaning | Ga^+^ ion | 30 | 2800 |
| 5 | Lamella cutting | Ga^+^ ion | 30 | 920 |
| 6 | Attachment to micromanipulator | Ga^+^ ion | 30 | 280 |
| 7 | Attachment to sample grid | Ga^+^ ion | 30 | 28 |
| 8 | Thinning from 2 μm to 1 μm | Ga^+^ ion | 8 | 110 |
| 9 | Thinning from 1 μm to 500 nm | Ga^+^ ion | 8 | 62 |
| 10 | Thinning from 500 nm to 200 nm | Ga^+^ ion | 8 | 21 |

**Cathodoluminescence and data processing in LumiSpy**

*Cross-sectional CL on lamella:* Cathodoluminescence (CL) hyperspectral maps were acquired in an Attolight Allalin 4027 Chronos CL-SEM, with the lamella oriented perpendicular to the electron beam path. The spectrum images were acquired with an iHR320 spectrometer (focal length of 320 mm, 150 gratings per mm blazed at 500 nm, 7000 µm entrance slit) and an Andor 1024 px charge-coupled device (4x horizontal binning and 1x signal amplification). A pulsed electron beam was used to acquire both CL maps of the FIB lamellae, at low beam currents to minimise specimen damage. This pulsed beam was obtained by pulsing an electron gun with the third harmonic of an Nd:YAG laser (λ = 355 nm) at a pulse width of 7 ps and a frequency of 80.6 MHz. All measurements were performed at room temperature under high vacuum. Beam focusing before each data acquisition was performed away from the sample areas used for the measurements to prevent specimen damage. CL maps were acquired using 5 kV acceleration voltage, 23 pA beam current, 124.1 ms/pixel acquisition time, and a pixel size of 277 nm, resulting in an electron dose of 2.32 e^-^/Å^2^. These beam parameters were found to be the optimum to minimise detrimental beam damage-related effects in the perovskite emission.

*Top-view CL on half-device:* The cross-sectional CL emission was compared to the emission from a reference half-PSC device with the same perovskite composition. The top-view CL map was acquired with the same instrument in continuous wave mode, at 5 kV acceleration voltage, 62.5 pA beam current, 48.8 ms/px acquisition time, and a pixel size of 284 nm, resulting in an electron dose of 2.36 e^-^/Å^2^. The horizontal binning and signal amplification parameters are the same as for cross-sectional CL. The luminescence spectra acquired from similar triple-cation double-halide perovskite films through continuous wave and pulsed mode CL are comparable to one another in terms of emission wavelength/energy, but not emission intensity.^1^

CL maps were processed and analysed in LumiSpy 0.1.^2^ Artefacts caused by cosmic rays saturating the spectrometer were removed, and the edges of each map were cropped out as they tend to show edge effects and higher CL intensities. The CL spectrum ($s$) in each pixel ($x$) was fitted to a linear summation of two Gaussian distributions ($g$) and a constant background offset ($k_{bkg}$):

$s\left( x \right)= k_{bkg}(x)+ g_{perovskite}\left( x \right)+ g_{PbI_{2}}\left( x \right)$ (Equation S1)

One Gaussian represents the perovskite’s emission and the other represents the emission of PbI_2_. Fitting was performed using least-squares optimization with the Levenberg-Marquardt algorithm.^3,4^

**Photoluminescence**

The PL data was acquired with a Photon Etc IMA microscope with a diffraction-limited spatial resolution (~500 nm with a 100× objective of numerical aperture = 0.9). A volume Bragg grating was placed before the camera to detect only specific wavelengths with a spectral resolution of 2.5 nm. A 405 nm laser was normally incident on the sample, with a spot size of ~150 µm in diameter. The sample stage was immobile during data acquisition while the collection wavelength was swept (integration time/wavelength = 3 s). The incident photon flux was equivalent to 1 sun illumination.


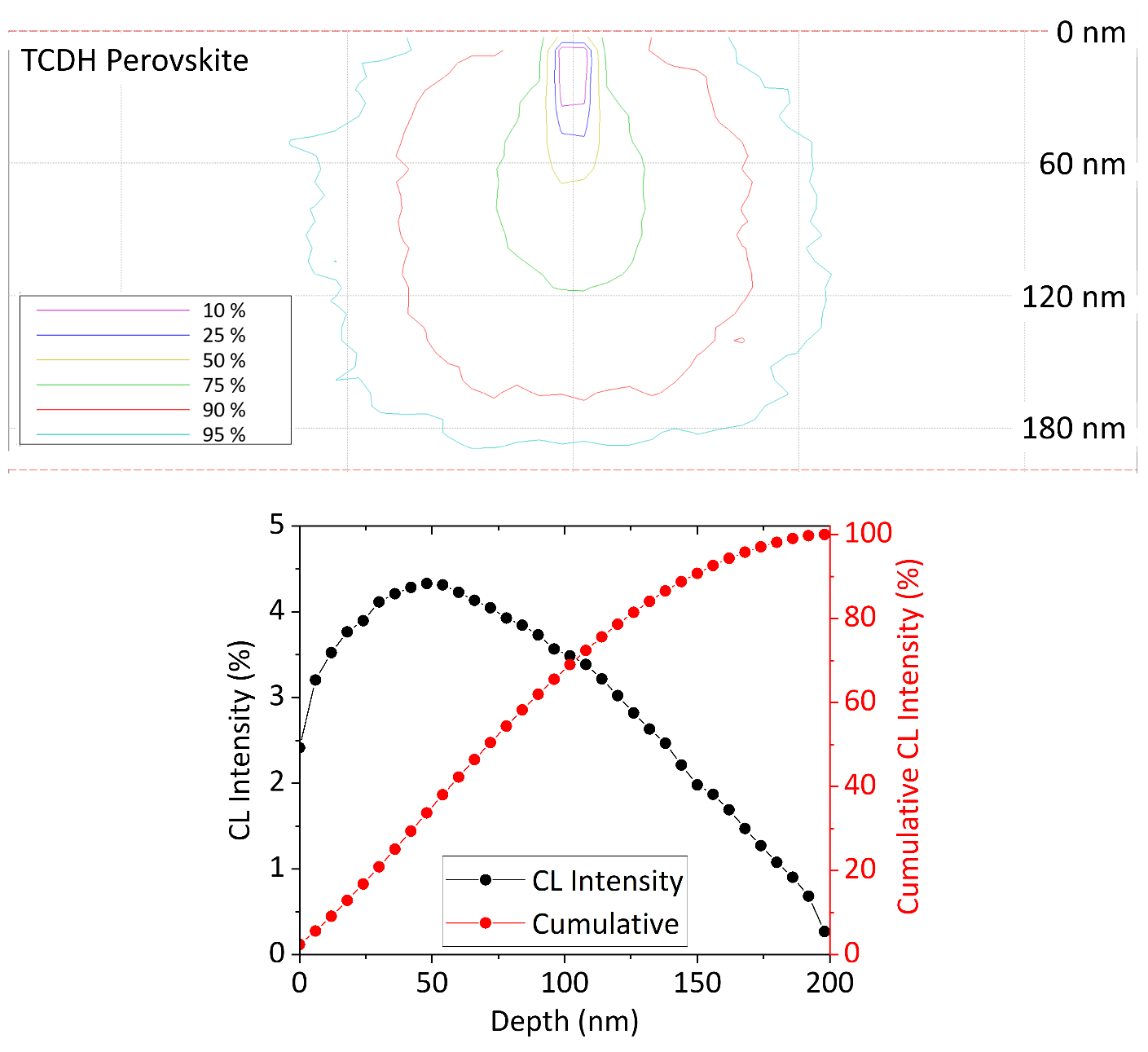


**Figure S1.** Result of Monte Carlo-based electron trajectory simulation using the CASINO software for an electron beam accelerating voltage of 5 kV and a model perovskite structure of composition Cs_0.05_FA_0.81_MA_0.14_Pb(I_0.9_Br_0.1_)_3_.^5^ The top panel shows the contours of energy deposition inside the 200 nm-thick perovskite layer, where each line marks a volume in which a certain portion of the electron beam’s energy was dissipated. The bottom panel shows the contribution of each thickness slice to the total detected CL signal (black curve) and the cumulative detected CL signal (red curve).


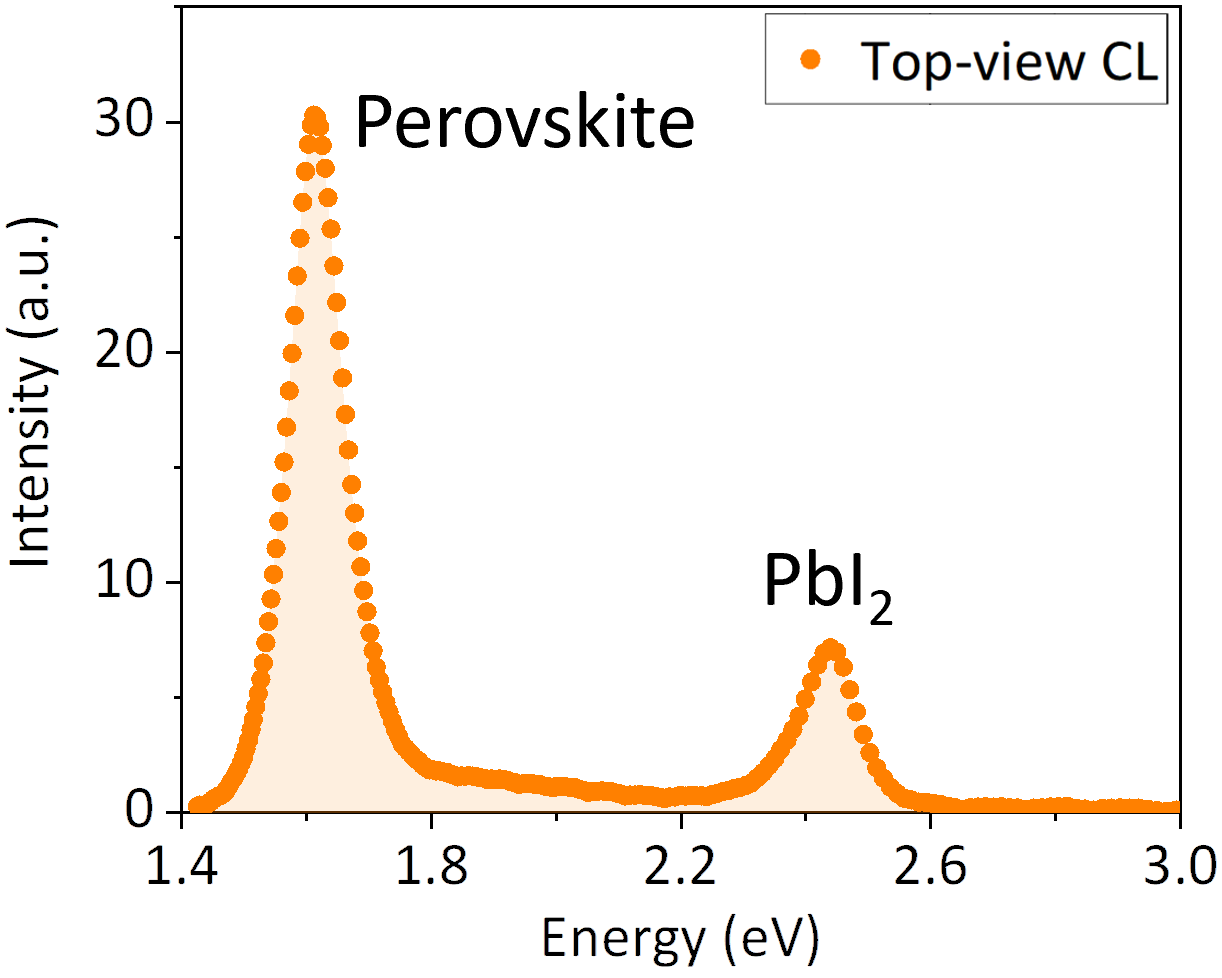


**Figure S2.** Perovskite and PbI_2_ emission spectrum from the top-view CL scan.

**Supplementary Note 1**

The relative contributions of the CL electron beam and the FIB Ga^+^ beam on the specimen amorphisation can be approximated by comparing the magnitude of the radiation pressure ($P$) they exerted on the specimen. $P$ is defined as the pressure imposed on a surface due to momentum transfer from an incident electromagnetic wave. It is calculated according to Equation S2, where $P$ is in Pa, $I$ is the incident wave’s intensity in W/m^2^, $c$ is the speed of light, and $\theta$ is the angle between the incident wave and the surface’s normal. An analogue of Equation S2 for a beam of electrically charged particles can be derived by calculating the beam’s intensity (Equation S3) and replacing $c$ with $v$, the particle velocity in m/s (Equation S4). In Equations S3 and S4, $i$ is the beam current in A, $V$ is the beam’s acceleration voltage in V, $d$ is the beam diameter in m, $v$ is the particle velocity in m/s, $q$ is the particle’s charge in C, and $m$ is the particle’s mass in kg. Furthermore, since the Ga^+^ beam is always brought back to focus every time the stage tilt is changed, the beam area is constant at all values of $\theta$. Consequently, only one $\cos(\theta)$ term is required in the formula for $P$, to represent the component of the incident ion’s momentum that is perpendicular to the lamella surface. The final formula is shown in Equation S5.

$P= \frac{I{cos}^{2}\theta}{c}$ (Equation S2)

$I=\frac{Beam Power}{Beam Area}=\frac{iV}{\pi{(\frac{d}{2})}^{2}}$ (Equation S3)

$v= \sqrt{\frac{2qV}{m}}$ (Equation S4)

$P=\frac{4i cos(\theta)\sqrt{Vm}}{\pi d^{2}\sqrt{2q}}$ (Equation S5)

$P$ for the cross-sectional CL scans and the final lamella thinning step of FIB milling were calculated using the parameters shown in Table S2. For the sake of completeness, the $P$ exerted by the laser beam used in PL and the electron beam in top-view CL was also calculated. As shown in the last row of Table S2, $P$ for the lamella thinning step is 5x lower than the $P$ for cross-sectional CL scans due to the greater beam diameter and glancing angle used in lamella thinning. This comparison suggests that the role of the CL electron beam in causing the observed emission blue-shift is significant, though not necessarily 5x greater than that of the Ga^+^ beam. The $P$ values obtained in Table S2 are far lower than the hydrostatic pressures used to amorphise perovskite in refs. ^6–10^, which are in the order of GPa. However, this does not mean the relationship between perovskite structure and luminescence observed in those studies cannot be applied in this study. Rather, it is simply the amorphisation mechanism that is different. Instead of relying on a very high pressure to trigger a transition into an amorphous phase, the amorphisation in FIB milling is caused by accumulation of defects created by radiolysis and atomic displacement.^11–14^

**Table S2.** Radiation pressure calculation for PL, CL, and FIB milling.

| Known Parameters | PL  (laser beam) | Top-view CL  (e^-^ beam) | Cross-sectional CL  (e^-^ beam) | FIB Milling  (Ga^+^ beam) |
| --- | --- | --- | --- | --- |
| $i$ (A) | N.A. | 6.25 × 10^-11^ | 2.30 × 10^-11^ | 2.1 × 10^-11^ |
| $V$ (V) | N.A. | 5.00 × 10^3^ | 5.00 × 10^3^ | 8.00 × 10^3^ |
| $m$ (kg) | N.A. | 9.11 × 10^-31^ | 9.11 × 10^-31^ | 1.16 × 10^-25^ |
| $d$ (m) | N.A. | 5.00 × 10^-9^ | 5.00 × 10^-9^ | 3.00 × 10^-8^ |
| $\vert q\vert$ (C) | N.A. | 1.60 × 10^-19^ | 1.60 × 10^-19^ | 1.60 × 10^-19^ |
| $\theta$ (^o^) | 0 | 0 | 0 | 89 |
| $c$ (m/s) | 3.00 × 10^8^ | N.A. | N.A. | N.A. |
| $I$ (W/m^2^) | 1 × 10^3^ | N.A. | N.A. | N.A. |
| Calculated Parameters | PL  (laser beam) | Top-view CL  (e^-^ beam) | Cross-sectional CL  (e^-^ beam) | FIB Milling  (Ga^+^ beam) |
| $I$ (W/m^2^) | N.A. | 1.59 × 10^10^ | 5.86 × 10^9^ | 2.38 × 10^8^ |
| $v$ (m/s) | N.A. | 4.19 × 10^7^ | 4.19 × 10^7^ | 1.49 × 10^5^ |
| $\boldsymbol{P}$ **(Pa)** | **3.33 × 10^-6^** | **3.79 × 10^2^** | **1.40 × 10^2^** | **2.79 × 10^1^** |


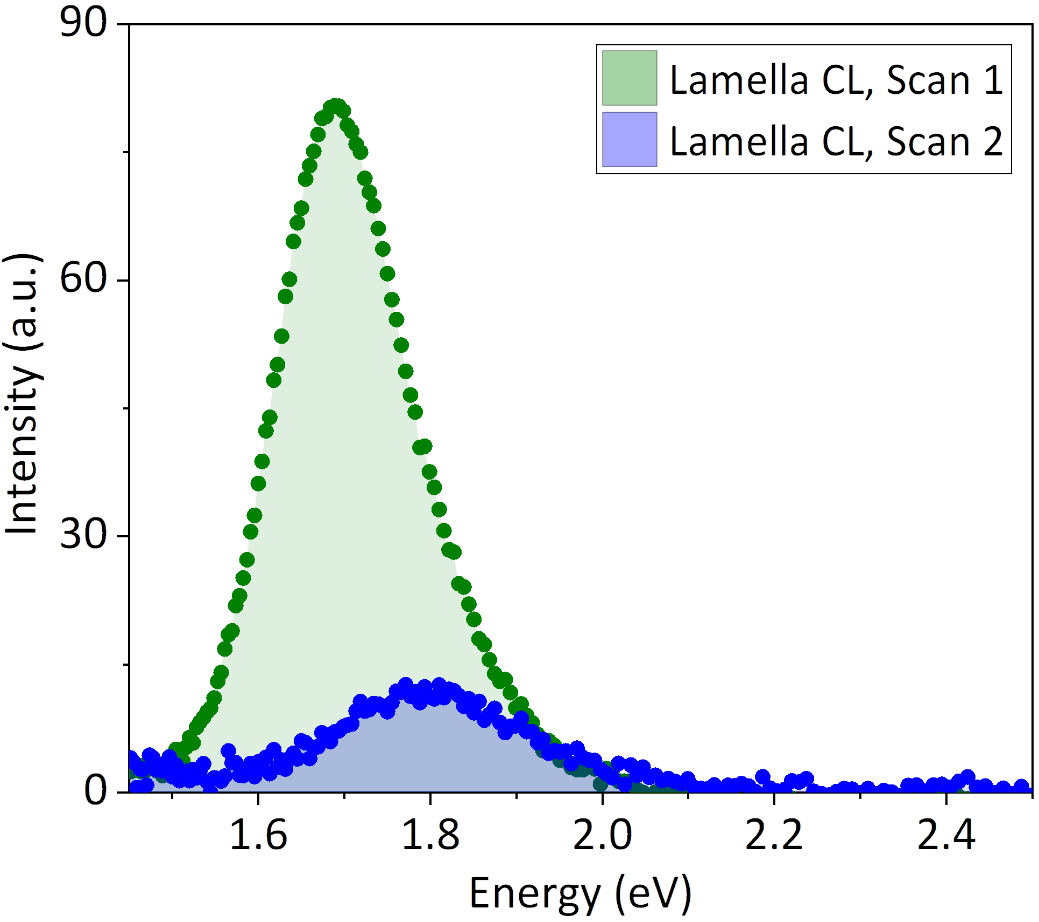


**Figure S3.** Perovskite emission spectra from (green) first cross-sectional CL scan and (blue) second cross-sectional CL scan. These spectra are averaged from all perovskite pixels in the CL spectrum images.

**References**

(1) Ferrer Orri, J.; Tennyson, E. M.; Kusch, G.; Divitini, G.; Macpherson, S.; Oliver, R.; Ducati, C.; Stranks, S. Using Pulsed Mode Scanning Electron Microscopy for Cathodoluminescence Studies on Hybrid Perovskite Films. *Nano Express* **2021**. https://doi.org/10.1088/2632-959X/abfe3c.

(2) Ferrer Orri, J.; Lähnemann, J.; Prestat, E.; Johnstone, D. N.; Tappy, N. LumiSpy. 2021. https://doi.org/10.5281/zenodo.4640446.

(3) Levenberg, K. A Method for the Solution of Certain Non-Linear Problems in Least Squares. *Q. Appl. Math.* **1944**, *2* (2), 164–168. https://doi.org/10.1090/qam/10666.

(4) Marquardt, D. W. An Algorithm for Least-Squares Estimation of Nonlinear Parameters. *J. Soc. Ind. Appl. Math.* **1963**, *11* (2), 431–441. https://doi.org/10.1137/0111030.

(5) Drouin, D.; Couture, A. R.; Joly, D.; Tastet, X.; Aimez, V.; Gauvin, R. CASINO V2.42—A Fast and Easy-to-Use Modeling Tool for Scanning Electron Microscopy and Microanalysis Users. *Scanning* **2007**, *29* (3), 92–101. https://doi.org/10.1002/sca.20000.

(6) Wang, Y.; Lü, X.; Yang, W.; Wen, T.; Yang, L.; Ren, X.; Wang, L.; Lin, Z.; Zhao, Y. Pressure-Induced Phase Transformation, Reversible Amorphization, and Anomalous Visible Light Response in Organolead Bromide Perovskite. *J. Am. Chem. Soc.* **2015**, *137* (34), 11144–11149. https://doi.org/10.1021/jacs.5b06346.

(7) Wang, P.; Guan, J.; Galeschuk, D. T. K.; Yao, Y.; He, C. F.; Jiang, S.; Zhang, S.; Liu, Y.; Jin, M.; Jin, C.; et al. Pressure-Induced Polymorphic, Optical, and Electronic Transitions of Formamidinium Lead Iodide Perovskite. *J. Phys. Chem. Lett.* **2017**, *8* (10), 2119–2125. https://doi.org/10.1021/acs.jpclett.7b00665.

(8) Zhu, H.; Cai, T.; Que, M.; Song, J. P.; Rubenstein, B. M.; Wang, Z.; Chen, O. Pressure-Induced Phase Transformation and Band-Gap Engineering of Formamidinium Lead Iodide Perovskite Nanocrystals. *J. Phys. Chem. Lett.* **2018**, *9* (15), 4199–4205. https://doi.org/10.1021/acs.jpclett.8b01852.

(9) Wang, L.; Wang, K.; Zou, B. Pressure-Induced Structural and Optical Properties of Organometal Halide Perovskite-Based Formamidinium Lead Bromide. *J. Phys. Chem. Lett.* **2016**, *7* (13), 2556–2562. https://doi.org/10.1021/acs.jpclett.6b00999.

(10) Zhang, L.; Zeng, Q.; Wang, K. Pressure-Induced Structural and Optical Properties of Inorganic Halide Perovskite CsPbBr_3_. *J. Phys. Chem. Lett.* **2017**, *8* (16), 3752–3758. https://doi.org/10.1021/acs.jpclett.7b01577.

(11) Huh, Y.; Hong, K. J.; Shin, K. S. Amorphization Induced by Focused Ion Beam Milling in Metallic and Electronic Materials. *Microsc. Microanal.* **2013**, *19* (S5), 33–37. https://doi.org/10.1017/S1431927613012282.

(12) Egerton, R. F.; Li, P.; Malac, M. Radiation Damage in the TEM and SEM. *Micron* **2004**, *35* (6), 399–409. https://doi.org/10.1016/j.micron.2004.02.003.

(13) Egerton, R. F. Radiation Damage to Organic and Inorganic Specimens in the TEM. *Micron* **2019**, *119*, 72–87. https://doi.org/10.1016/j.micron.2019.01.005.

(14) Baba, A.; Bai, D.; Sadoh, T.; Kenjo, A.; Nakashima, H.; Mori, H.; Tsurushima, T. Behavior of Radiation-Induced Defects and Amorphization in Silicon Crystal. *Nucl. Instruments Methods Phys. Res. Sect. B Beam Interact. with Mater. Atoms* **1997**, *121* (1–4), 299–301. https://doi.org/10.1016/S0168-583X(96)00392-8.
